# Reducing the Negative Effect on White Wine Chromatic Characteristics Due to the Oxygen Exposure during Transportation by the Deoxygenation Process

**DOI:** 10.3390/foods10092023

**Published:** 2021-08-28

**Authors:** Luís Filipe-Ribeiro, Susete Rodrigues, Fernando M. Nunes, Fernanda Cosme

**Affiliations:** CQ-VR, Chemistry Research Centre—Vila Real, Food and Wine Chemistry Lab., Universidade de Trás-os-Montes e Alto Douro, Apartado 1013, 5000-801 Vila Real, Portugal; fmota@utad.pt (L.F.-R.); susete_rodrigues@iol.pt (S.R.); fnunes@utad.pt (F.M.N.)

**Keywords:** white wine, oxygen, deoxygenation, phenolic compounds, chromatic characteristics

## Abstract

In white wine production, a great effort is made to avoid extensive contact with oxygen, which might adversely affect color and aroma. In this work, the impact of bulk transportation on white wine oxygen uptake and the effect of deoxygenation on white wine dissolved oxygen levels, as well on the phenolic composition and chromatic characteristics of white wines stored for nine months, were studied. Transportation increased the white wine dissolved oxygen content (117 and 181% in the wines studied) that increased the free sulfur dioxide loss during storage. Moreover, deoxygenation of white wines reduced the increase in the yellow color of white wines during storage, probably related to the higher levels of free sulfur dioxide that remain in these wines during storage. Furthermore, the amount of wine phenolics also have a decisive influence on wine color characteristics evolution, with increased levels of total phenolic compounds increasing the variation in the b *(measure of yellowness) values of the wines after nine months of storage. Results show the negative impact of bulk transportation on white wine color characteristics; however, wine deoxygenation is a good practice to minimize those aspects, preserving color characteristics.

## 1. Introduction

Oxygen management in wine production is important to guarantee the high quality since wine composition changes are highly dependent on the amount of dissolved oxygen [1,2] playing an important role in several winemaking processes [3]. However, when oxygen is present at certain critical stages or in higher levels than recommended, it can have a negative impact on wine quality, such as growth stimulation of undesirable microorganisms or undesirable changes in wine color and aroma [4].

During the different wine manipulation processes, oxygen dissolution can occur, as for example during wine transfer operations (racking, pumping), filtration, stabilization, or bottling [1,2,5,6].

White wine production involves a great effort to avoid extensive contact with oxygen, which might adversely affect the color and eventually the decline of the overall quality and marketability because the oxidation of substances can occur at any time during winemaking [7,8]. The oxidative degradation of white wines rapidly leads to a loss of their sensory quality [9]. From an aromatic point of view, this phenomenon leads to a loss of characteristic aromas of young wines, namely, the floral and fruity aromas, and subsequently leads to the formation of new aromas characteristic of older wines or atypical aromas associated with the degradation of the product [10,11,12,13]. In bottled white wines, it is recognized that the aromatic decline occurs prior to the chromatic changes [7,12].

It is well established that the relevant reactions that lead to browning development in white wines are mainly dependent on the wine polyphenolic composition [14,15,16,17].

The solubility of oxygen from the air into wine saturated at atmospheric pressure is about 6 to 8 mg/L, depending on wine composition and temperature [8,18]. Phenolic interactions (i.e., their oxidation and polymerization) are initiated and are further promoted by the absorption of oxygen by wine, even if the rate of progression from monomeric to polymeric pigment forms depends largely upon temperature [19]. Oxygen absorption rate depends on the oxidative mechanisms: enzymatic and auto-catalytic reactions; tannin polymerization; anthocyanin–tannin direct reactions which are mediated by acetaldehyde condensation [20]. When ascorbic acid is used in white wines for delaying browning, it can be oxidized to preference polyphenols, generating dehydroascorbic acid and hydrogen peroxide [21]. However, hydrogen peroxide was considered to be more active than oxygen and could cause further oxidation [22].

Under wine oxidation conditions, phenolic compounds are one of the primary reactants with oxygen, mainly with reactive species of oxygen which are the activated oxygen species formed during oxygen reduction in the presence of metals [18,23]. Oxygen management represents a major challenge in enology as both excessive exposure and excessive protection lead to sensory defects [4,9]. Sulfur dioxide may limit chemical oxidation reactions by reducing the oxidation products to their original form (*o*-quinones causing their reduction to the original phenolic compound) or may react with hydrogen peroxide (resulting in sulfate (VI) and water) or forming a product with a sulfone group [24]. However, the reaction rate of oxygen with sulfur dioxide is quite slow relative to that which can occur in wine, and it has been argued that the main antioxidant action of sulfur dioxide is through the reaction with hydrogen peroxide produced as a result of polyphenol oxidation [25,26]. However, several studies have focused their attention on the effect of oxygen on wine characteristics but there are very few works on the effect of wine deoxygenation.

There is empirical evidence that wine bulk transportation reduced its quality by increasing oxygen exposure; nevertheless, there is no deep knowledge to what extent it contributes to the wine oxygen uptake. Furthermore, there are no studies concerning preventive measures including the deoxygenation process, its efficiency, and impact on wine color characteristics. Therefore, the purpose of this work was to study the effect of wine bulk transportation on wine oxygen uptake and the efficiency of wine deoxygenation on the reversion of the negative impacts on wine color characteristics of oxygen uptake during transportation. The goal of this research was to monitor only the parameters influencing the chromatic characteristics of the wines. The olfactory sensation of “oxidation” for these wines and the change in volatile compounds has not been evaluated. As far as we know, this is the first work that studies the efficiency of the deoxygenation process as a preventive measure to cope with the negative impact of oxygen uptake in wine color characteristics during wine bulk transport.

## 2. Materials and Methods

### 2.1. Wine Samples

Two young white wines from the Vinho Verde region bottled 3 months after harvest were used (northwest Portugal) to study the impact of the transportation and deoxygenation process during 9 months of bottle storage. The chemical characteristics of the monovarietal wine (Loureiro grape variety) and Blend wine were, respectively, as follows: pH 2.91 and 3.36; alcohol content (% *v/v*) 9.0 and 11.9; titratable acidity (g/L tartaric acid) 10.3 and 5.7; density at 20 °C (g/mL) 0.9960 and 0.9919. The wines were collected in triplicate in three different stages, before transportation (Stage 0), after transportation (Stage 1), and after deoxygenation (Stage 2). The physicochemical analysis was performed in triplicate at the initial time (T0), first month (T1), third month (T3), sixth month (T6), and ninth month (T9). The sample code provides three different pieces of information: Wine: B—Blend wine, L—Loureiro monovarietal wine; Stage: 0—before transportation, 1—after transportation, 2—after deoxygenation process; Time: T0 (initial time), T1 (first month), T3 (third month), T6 (sixth month), and T9 (ninth month).

### 2.2. Experimental Design

For the deoxygenation process, the wine was submitted to continuous nitrogen (purity > 99.5%) diffusion from the transport cistern vat to the wine vessel. The amount of nitrogen injected was about 10% of the transfer pump flow according to the manufacturer instructions (IOC ENOTECNIA, www.enotecniaioc.com (accessed on: 30 June 2021). They were stored in 750 mL glass bottles (bordelaise prestige, antique color) with a headspace around 6 mm and with a free sulfur dioxide at 40 mg/L. The bottles were closed with a cork (Twin Top) due to the low rate of oxygen diffusion [27] during the study over 9 months. The samples were placed for 12 h in a vertical position and stored for nine months in the horizontal position at 20 °C.

### 2.3. Analysis of Conventional Enological Parameters

Alcohol content (method OIV-MA-AS312-01B), total acidity (method OIV-MA-AS313-01), volatile acidity (method OIV-MA-AS313-02), fixed acidity (method OIV-MA-AS313-03), pH (OIV-MA-AS313-15), free and total sulfur dioxide (OIV-MA-AS323-04B), and density at 20 °C (method OIV-MA-AS2-01B) were measured according to standard methods of Organization International de la Vigne et du Vin [28]. 

### 2.4. Total Phenols, Non-Flavonoid, and Flavonoid Phenols

Determination of the phenolic content of wines was carried out by absorbance measurement at 280 nm before and after precipitation of the flavonoids through reaction with formaldehyde according to Kramling and Singleton [29], leading to the quantification of non-flavonoid compounds in the wine. The results were expressed as gallic acid equivalents/L.

### 2.5. Total Tannins

The total tannin content was determined according to Ribéreau-Gayon and Stonestreet [30].

### 2.6. Gelatin Index

The gelatin index of tannins was measured by the method proposed by Glories [31] using 50 mL of wine and 5 mL of a gelatin solution (70 g/L).

### 2.7. Color and Chromatic Characteristics

The color was determined by measuring absorbance at 420 nm (10 mm cell) according to OIV [28]. The wine chromatic characteristics were determined by the absorption spectra of the wine samples scanned over the range 380–780 nm using quartz cells of 1 cm path length. Data were collected at 10 nm intervals, and referenced to 1 cm path length, to calculate L * (lightness), a * (measure of redness), b * (measure of yellowness); coordinates using the CIEL*a*b* method [28]. The spectrophotometer incorporates the software required to calculate the CIEL*a*b* parameters directly. To differentiate the color more precisely, the color difference was obtained using the following expression: ΔE * = [(ΔL *)^2^ + (Δa *)^2^ + (Δb *)^2^]^1/2^. It quantifies the overall color difference of a sample when compared to a reference sample (wine before transport). Two colors can be distinguished by the human eye when the difference between ΔE * values is greater than 2 units [32].

### 2.8. Acetaldehyde

Determination of acetaldehyde concentrations was performed using the spectrophotometric enzyme assay kit (Boehringer, R-Biopharm, Germany). This consists of oxidation of acetaldehyde to acetic acid in the presence of aldehyde dehydrogenase (AI-DH) in the presence of nicotinamide adenine dinucleotide (NAD+). The amount of nicotinamide adenine dinucleotide hydride (NADH), which is proportional to the amount of acetaldehyde, is determined by observance at 340 nm.

### 2.9. Dissolved Oxygen

Dissolved oxygen was quantified using a selective membrane electrode (OXI 45P, CRISON). The oxygen diffuses through the permeable membrane and reacts at the cathode generating a proportional current to the concentration of dissolved oxygen (DO). The equipment transforms this current in mg/L of DO.

### 2.10. Kinetic Analysis—Kinetic Modeling

In order to model the kinetics of the variation of dissolved oxygen levels, free sulfur dioxide, volatile acidity, color, and total phenols were calculated by integrating the differential Equation (1).

(1) $$\frac{d\left[A\right]}{dt}=-k_{eff}{\left[A\right]}^{a}$$

Thus, for zero (*a* = 0), first (*a* = 1), and second (*a* = 2) order reactions, the result is Equations (2)–(4), respectively.

(2) $$[A{]}_{t}=[A{]}_{0}-k_{eff}\left(t-t_{0}\right)$$

(3) $$ln[A{]}_{t}=ln[A{]}_{0}-k_{eff}\left(t-t_{0}\right)$$

(4) $$\frac{1}{{\left[A\right]}_{t}}=\frac{1}{\;{\left[A\right]}_{0}}-k_{eff}\left(t-t_{0}\right)$$

In Equations (2) and (3), [*A*]*_t_* and [*A*]_0_ represent the level of the compound or chromatic parameter at time *t* and *t*_0_, respectively. To determine the reaction order, the integrated kinetic equations of zero, first, and second order were tested to find the reaction order that best fit the experimental data. The correlation coefficient and the residue distribution were both used to evaluate the experimental values’ adequacy to the different kinetic models [33]. To check whether there were differences between the slopes (rate constants), Fisher’s least significant difference (LSD) test was used.

### 2.11. Statistical Analysis

The data are presented as means ± standard deviation. Physicochemical data were statistically tested by analysis of variance (one-way ANOVA) using the Statistica 7 software (StatSoft, Tulsa, OK, USA) program. Tukey’s honestly significant difference (HSD, 5% level) test was applied to the physicochemical data to determine significant differences between the parameters. Levene’s test was used for testing the homogeneity of variances and no significant differences were found.

## 3. Results and Discussion

### 3.1. Effect of Oxygen Uptake during Transportation and Deoxygenation on Wine Quality

#### 3.1.1. Oxygen, Sulfur Dioxide, Acetaldehyde, Volatile Acidity Levels during Storage

As can be observed in Table 1, the transport (Stage 0 to Stage 1, at the initial time T0) of the two wines studied resulted in a significant increase in wine dissolved oxygen levels (Loureiro monovarietal wine increased by 181% (2.71 mg/L) and Blend wine increased by 117% (2.32 mg/L)). Under our experimental conditions, transportation always increased the concentrations of dissolved oxygen; these results agree with previous studies conducted by Vidal et al. [5]. The difference in the extent of increase observed for the oxygen levels between both wines can be explained by the lower dry extract of Blend wine (24.5 g/L) when compared to the Loureiro monovarietal wine (26.1 g/L). In general, the salts and sugars cause an increase in Henry’s constant with respect to that of water and therefore a decrease in oxygen solubility [34]. During storage (initial time—T0 till ninth month—T9), there was observed a decrease in the dissolved oxygen levels for the two wines before (Stage 0) and after transportation (Stage 1) (Table 1). The decrease of dissolved oxygen levels during storage of the wines before (Stage 0) and after transportation (Stage 1) followed a second-order kinetic model (Table 2). However, the deoxygenation process with nitrogen lowered the dissolved oxygen by 4.12 and 4.21 mg/L for the Loureiro monovarietal wine and the Blend wine, respectively, reaching low levels of dissolved oxygen around 0.09 mg/L at the initial time (T0) after the deoxygenation process (Stage 2) in both wines (T0L2 and T0B2, Table 1). These low levels of dissolved oxygen were maintained with a slight reduction during storage (initial time—T0 till ninth month—T9) of the wines with deoxygenation (Stage 2). The dissolved oxygen level after nine months (T9) of wine storage with deoxygenation was 0.07 mg/L for the Loureiro monovarietal wine and 0.02 mg/L for the Blend wine that were significantly lower when compared to the wine after transport (Stage 1) and also when compared with the initial wine without transportation (Stage 0, Table 1). 

As shown in Table 1, after transportation (Stage 1) the level of free sulfur dioxide decreased significantly, but at the same time, the dissolved oxygen increased significantly, which could explain the decrease observed in the levels of free sulfur dioxide for these wines. These results are in accordance with Jacobs [35], who verified a relation between the decrease of free sulfur dioxide and the presence of dissolved oxygen. This decrease of sulfur dioxide is not related to the direct interaction of oxygen with sulfur dioxide but due to the oxidation of sulfur dioxide through a radical chain reaction [26,36]. The decrease of free sulfur dioxide was higher in the first month (T1), which could be related to the higher levels of dissolved oxygen in this step (Table 1); these results are in agreement with Wirth et al. [37] who also verified a relation between wine oxygen exposure and sulfite consumption. Moreover, the free sulfur dioxide level decrease in both wines during bottle storage (nine months), following a second-order kinetic model (Table 2). For the Blend wine, the increase in dissolved oxygen level resulting from transportation (Stage 0 to Stage 1) increases the rate of oxygen consumption (3.28×). Furthermore, the increase in dissolved oxygen level increased on average the rate of free sulfur dioxide consumption for the Blend wine (1.45×). For the Blend wine, there was a significant correlation between the loss of dissolved oxygen levels and the decrease in free sulfur dioxide levels; nevertheless, the slopes were significantly different for the consumption of dissolved oxygen before (Stage 0) and after transportation (Stage 1), with the consumption of dissolved oxygen before transportation (Stage 0) showing a higher slope than after transportation (Stage 1). This means that for each milligram of dissolved oxygen per liter there was a significantly higher consumption of free sulfur dioxide. These results are in accordance with the literature [38]. For the Loureiro monovarietal wine, a different behavior was observed (Table 2). The apparent kinetic second-order constant decreased when the amount of dissolved oxygen increased (Table 2). These results can be due to the fact that previous oxygen dissolution history from both wines were unknown, and according to Carrascon et al. [39], the rate of oxygen consumption in white wines tends to decrease with increasing cycles of oxygen dissolution and consumption. Moreover, the lower number of total phenols in Loureiro monovarietal wine when compared to Blend wine can explain these differences, as when oxygen is dissolved in wine, it is quickly consumed, as it is involved in numerous mechanisms of reduction–oxidation reactions, with phenolic compounds being the main consumers of oxygen (around 60%) [40]. Nevertheless, the rate constants for oxygen consumption for the higher dissolved oxygen levels found in the wine after transportation (Stage 1) for Loureiro monovarietal wine and for Blend wine were not significantly different (Table 2). 

Furthermore, for Loureiro monovarietal wine, the pseudo-second-order rate constants of free sulfur dioxide consumption were higher than that observed for the Blend wine. For Blend wine there was observed a linear correlation between the dissolved oxygen decrease and sulfur dioxide decrease (r = 0.986, *p* < 0.0022, and r = 0.997, *p* < 0.00020 for wine before—Stage 0 and after transportation—Stage 1, respectively). Contrarily to that observed for the Blend wine, for Loureiro monovarietal wine there was not observed a linear but a negative correlation between the free sulfur dioxide consumption and the inverse of oxygen consumption (r = 0.965, *p* < 0.0078, and r = 0.992, *p* < 0.0008600020 for wine before—Stage 0 and after transportation—Stage 1, respectively). This lack of direct relation between the decrease of sulfur dioxide with the decrease in dissolved oxygen can be explained, as mentioned previously, by the fact that sulfur dioxide consumption is not related to the direct interaction of oxygen with sulfur dioxide but due to the oxidation of sulfur dioxide through a radical chain reaction [36]. The deoxygenation process allowed us after the 9-month storage period to obtain a free sulfur dioxide level significantly higher than that observed for the wine after transportation (Stage 1) and identical or higher than the wine before transportation (Stage 0) for the Blend and Loureiro monovarietal wines, respectively. The second-order apparent rate constant for the free sulfur dioxide decrease in the wines after deoxygenation when compared to the wine after transportation (Stage 1) for Loureiro monovarietal wine (0.67×) and Blend wine (0.43×) and also when compared to the wine before transportation (Stage 0; 0.62× and 0.63× for Loureiro monovarietal wine and Blend wines, respectively; Table 2).

These results show that the behavior of the two wines during oxidation was very different. In fact, for Loureiro monovarietal wine during the 9-month period, the levels of acetaldehyde did not increase significantly for the wine before (Stage 0) and after transportation (Stage 1), but for the Blend wine there was observed a significant increase in the concentration of acetaldehyde during storage (initial time—T0 till ninth month—T9), although not significantly different for the wine before (Stage 0) and after transportation (Stage 1). The results obtained for Loureiro monovarietal wine are identical to the results obtained by Escudero et al. [41] and Carlton et al. [42]. Oxygen oxidizes phenolic compounds, resulting in hydrogen peroxide generation, which in turn oxidizes ethanol to acetaldehyde [3].

Volatile acidity increased linearly with time (nine months) (Table 2) during bottle storage for both wines before transportation (Stage 0) with a rate of increase not significantly different between the Loureiro monovarietal wine (0.28 to 0.39 g/L) and Blend wines (0.31 to 0.44 g/L). After transportation (Stage 1), there was also observed a linear increase in the volatile acidity of both wines, with a rate not significantly different before transportation (Stage 0) and between both wines (Table 1). In opposition, the deoxygenated wine (Stage 2) showed a lower increase in volatile acidity for Loureiro monovarietal wine and identical increase for Blend wine (Table 1). In the deoxygenated Loureiro monovarietal wine, a significant increase in volatile acidity was only detected after nine months (T9L2), and after six months for the Blend wine (T6B2). However, for the non-deoxygenated Loureiro monovarietal wine (Stage 1), a significant increase in volatile acidity was observed just after the third month. The rate of volatile acidity increase was significantly lower for the deoxygenated wine (Stage 2) when compared to the wines before transport (Stage 0), although not significantly lower than the rate observed for the wines stored after transport (Stage 1). The decrease of the zero-order rate constant for the evolution of volatile acidity after deoxygenation (Table 2) might be explained by the fact that higher oxygen levels in wine can favor the activity of harmful microorganisms which develop under aerobic conditions; for example, acetic acid bacteria [3].

#### 3.1.2. Color and Chromatic Characteristic Evolution during Storage

The color of white wines is one of the main color characteristic parameters of this product. The appearance of a brown color in white wines that are commercialized in white bottles is one of its limiting color characteristic parameters, namely, exhibiting non-acceptable color. The combination of lighter and/or thinner bottles may offer less light protection and have potential major implications for wine visual color stability [43]. Therefore, the chromatic characteristics of the white wine were measured before (Stage 0) and after transportation (Stage 1), as well as during the nine months of storage (initial time—T0 till ninth month—T9) (Table 3). White wine browning is the result of a complex sequence of oxidation reactions that could occur during processing, aging, or storage [10]. The presence of high concentrations of phenolic compounds, low levels of free sulfur dioxide, and other reducing substances such as ascorbic acid increases the wine susceptibility to oxidation [44,45]. As can be observed in Table 3, the b * value (measure of yellowness) of the Blend wine increased significantly in transportation after the 9-month storage period (Stage 0 to Stage 1, T9), probably due to the higher exposure to oxygen. The difference in the b * value after the nine-month storage period for the wine after transportation (Stage 1) when compared to the wine before transportation (Stage 0) was higher for the Blend wine when compared to the Loureiro monovarietal wine. This different behavior can be related to the different phenolic composition of both wines (discussed below). The deoxygenation process (Stage 2) improved significantly the resistance of white wine to increase the b * value (Table 3). For Loureiro monovarietal wine, deoxygenation (Stage 2) of the wine after transportation (Stage 1) resulted in the preservation of the b * value, as there was no significant increase in this parameter. For the Blend wine, although a yellowing was observed, it was significantly lower than that observed after transportation (Stage 1) and even before transportation (Stage 0). According to Spagna et al. [32], if the values for the color difference (ΔE *) are lower than two units, it could not be discriminated visually. The values obtained for the Loureiro monovarietal wine indicated that color difference cannot be distinguished visually after nine months of storage (Stage 0, Stage 1, and Stage 2). In the Blend wine, according to these analytical data, it might be possible to distinguish visually the deoxygenated wine in relation to the non-deoxygenated wine after six and nine months of storage (T6B and T9B). The increase in the b * value during storage followed a zero-order kinetic model (Table 4), and although for the Loureiro monovarietal wine there was not observed a significant increase in the rate constant after transportation (Stage 1), for Blend wine there was observed a significant increase in the rate constant of yellowing (5.65×). The deoxygenation process allowed a significant decrease in the zero-order rate constant of wine yellowing for both wines (0.55× and 0.63× for Loureiro and Blend wines, respectively; Table 4).

#### 3.1.3. Effect of Oxygen Content on the Phenolic Composition Evolution during Storage

In general, no significant differences were observed for total phenols, non-flavonoid and flavonoid compounds, and total tannins during wine transportation (Stage 0 to Stage 1) in the two wines studied (Table 5). There was observed a significant decrease in the levels of total phenols, non-flavonoid phenols, and flavonoid phenols in both wines after storage for nine months. The levels of total phenols after nine months of storage were significantly higher after deoxygenation (Stage 2) when compared to the wine after transportation (Stage 1) for Blend wine, but no significant differences were observed in the levels of total phenols after nine months of storage for the Loureiro monovarietal wine (Table 5). The decrease verified in total phenolic compounds during the storage period (initial time—T0 till ninth month—T9) is in agreement with previous studies [46]. The decrease of flavonoid compounds during the storage time (nine months, Table 5) was similar to the results obtained by Pérez-Magariño and González-San José [47] in white wine stored for one year.

For Blend wine, the loss of total phenols followed a zero-order kinetic model (Table 6), and the rate constant of phenolic compound loss during storage before (Stage 0) and after transportation (Stage 1) and deoxygenation (Stage 2) were not significantly different for Loureiro monovarietal wine. For Blend wine, transportation (Stage 1) increases the rate constant of phenolic compound loss when compared to the initial wine; nevertheless, deoxygenation was not able to reduce the rate constant of phenolic compound loss during storage (Table 6). This different behavior between Loureiro monovarietal and Blend wines can be related to the different levels of phenolic compounds of these two wines. Blend wine presenting 1.40 times higher levels of phenolic compounds when compared to Loureiro monovarietal wine probably will have a higher antioxidant capacity, delaying the oxidation in Stage 0 (Table 5), and therefore when the amount of dissolved oxygen increases no difference is observed for Loureiro monovarietal wine. However, for the Blend wine, the oxidation of phenolic compounds is forced by the increase in the wine dissolved oxygen levels. The deoxygenation process was not able to delay the phenolic compound loss as the oxidation mechanism was already initiated in Stage 1 when the oxygen uptake takes place, and was not inhibited by the oxygen removal as the radical mechanism had already started.

The Loureiro monovarietal wine showed higher values of gelatin index than the Blend wine, which indicates the presence of more reactive tannins with proteins [7]. However, the values of the gelatin index decreased during the nine months in both wines, which could probably be related to tannin polymerization reactions during storage [48].

## 4. Conclusions

Transportation increases the white wine dissolved oxygen content that increases the free sulfur dioxide loss during storage (second-order rate constant before transportation: 0.00353 ± 0.00014 and 0.00081 ± 0.00011; and after transportation: 0.00325 ± 0.00024 and 0.00118 ± 0.00024, for Loureiro monovarietal and Blend wine, respectively). The deoxygenation process is an efficient process of decreasing the dissolved oxygen content of white wines and to decrease the loss of free sulfur dioxide content of wines during storage (0.00218 ± 0.00010 and 0.000510 ± 0.00012, for Loureiro monovarietal and Blend wine, respectively). Moreover, deoxygenation of white wines reduced the increase in the yellow color of white wines during storage, probably related to the higher levels of free sulfur dioxide that remain in these wines during storage (pseudo-zero-order rate constant for b * value increase after transportation: 0.253 ± 0.030 and 0.616 ± 0.068; and after deoxygenation: 0.0122 ± 0.0278 and 0.256 ± 0.058, for Loureiro monovarietal and Blend wines, respectively). Furthermore, the amount of wine phenolics have also a decisive influence on wine color characteristics evolution, with increased levels of total phenolic compounds increasing the variation in the b * values of the wines after nine months of storage. The outcomes of this study point out clearly the negative impact of white wine bulk transportation on the wine color characteristics due to the increase in dissolved oxygen levels; nevertheless, wine nitrogen deoxygenation is a good treatment to decrease these negatives aspects, decreasing the rate of yellowness of white wines. Further studies are needed to fully evaluate the impact of the deoxygenation process on the quality of white wines, especially on the sensory quality as it is known that degradation of the aroma also occurs, normally with the appearance of cooked-vegetable odor nuances.

## Figures and Tables

**Table 1:** Volatile acidity, free sulfur dioxide, dissolved oxygen, and acetaldehyde of the Loureiro monovarietal wine and the Blend wine before and after transportation and deoxygenation over nine months (mean ± standard deviation).

		Volatile Acidity (g/L Expressed as Acetic Acid)	Free Sulfur Dioxide (mg/L)	Dissolved Oxygen (mg/L)	Acetaldehyde (mg/L)		Volatile Acidity (g/L Expressed as Acetic acid)	Free Sulfur Dioxide (mg/L)	Dissolved Oxygen (mg/L)	Acetaldehyde (mg/L)
	Loureiro Monovarietal Wine					Blend Wine				
Initial time	T0L0	0.28 ± 0.01 ^a^	44 ± 1 ^a^	1.50 ± 0.04 ^a^	29.8 ± 1.0 ^a^	T0B0	0.31 ± 0.01 ^a^	41 ± 1 ^a^	1.98 ± 0.09 ^a^	34.5 ± 1.3 ^a^
	T0L1	0.29 ± 0.01 ^a^	39 ± 1 ^b^	4.21 ± 0.04 ^b^	29.3 ± 0.5 ^a^	T0B1	0.34 ± 0.02 ^a^	39 ± 0 ^a^	4.30 ± 0.01 ^b^	33.5 ± 0.6 ^a^
	T0L2	0.28 ± 0.01 ^a^	37 ± 1 ^c^	0.09 ± 0.01 ^c^	29.0 ± 0.0 ^a^	T0B2	0.35 ± 0.00 ^a^	37 ± 0 ^a^	0.09 ± 0.01 ^c^	32.3 ± 0.5 ^a^
First month	T1L0	0.29 ± 0.01 ^a^	35 ± 1 ^d^	0.36 ± 0.02 ^d^	31.0 ± 0.8 ^a^	T1B0	0.33 ± 0.02 ^a^	36 ± 1 ^a^	1.23 ± 0.02 ^d^	37.3 ± 0.5 ^b^
	T1L1	0.29 ± 0.00 ^a^	31 ± 1 ^e^	0.66 ± 0.03 ^e^	28.3 ± 0.5 ^a^	T1B1	0.38 ± 0.01 ^a^	29 ± 1 ^b^	1.11 ± 0.01 ^e^	34.8 ± 0.5 ^a^
	T1L2	0.28 ± 0.01 ^a^	32 ± 0 ^e^	0.07 ± 0.01 ^c^	29.0 ± 0.0 ^a^	T1B2	0.37 ± 0.02 ^a^	31 ± 1 ^c^	0.07 ± 0.02 ^c^	36.8 ± 1.5 ^b^
Third month	T3L0	0.31 ± 0.01 ^a^	26 ± 1 ^f^	0.25 ± 0.03 ^f^	31.8 ± 0.5 ^a^	T3B0	0.35 ± 0.01 ^a^	34 ± 2 ^c^	0.98 ± 0.01 ^f^	38.5 ± 1.3 ^b^
	T3L1	0.34 ± 0.01 ^b^	25 ± 0 ^f^	0.42 ± 0.02 ^g^	33.0 ± 1.0 ^a^	T3B1	0.44 ± 0.01 ^b^	27 ± 1 ^b^	0.81 ± 0.01 ^g^	34.5 ± 3.7 ^a^
	T3L2	0.31 ± 0.02 ^a^	30 ± 0 ^e^	0.08 ± 0.01 ^c^	30.3 ± 0.1 ^a^	T3B2	0.37 ± 0.01 ^a^	31 ± 1 ^c^	0.05 ± 0.01 ^c^	36.8 ± 1.7 ^b^
Sixth month	T6L0	0.34 ± 0.01 ^b^	22 ± 0 ^g^	0.15 ± 0.01 ^c^	29.8 ± 1.0 ^a^	T6B0	0.42 ± 0.01 ^b^	32 ± 1 ^c^	0.91 ± 0.01 ^f^	45.5 ± 1.3 ^c^
	T6L1	0.37 ± 0.01 ^c^	20 ± 1 ^g^	0.27 ± 0.01 ^f^	30.0 ± 0.0 ^a^	T6B1	0.45 ± 0.01 ^b^	26 ± 1 ^b^	0.42 ± 0.04 ^h^	42.3 ± 0.5 ^c^
	T6L2	0.33 ± 0.01 ^a^	26 ± 0 ^f^	0.08 ± 0.01 ^c^	31.0 ± 0.0 ^a^	T6B2	0.43 ± 0.01 ^b^	30 ± 1 ^c^	0.03 ± 0.01 ^c^	31.0 ± 0.0 ^a^
Ninth month	T9L0	0.39 ± 0.01 ^c^	18 ± 0 ^g^	0.12 ± 0.01 ^c^	29.5 ± 0.6 ^a^	T9B0	0.44 ± 0.01 ^b^	31 ± 1 ^c^	0.58 ± 0.01 ^i^	46.3 ± 1.0 ^d^
	T9L1	0.37 ± 0.01 ^c^	17 ± 1 ^g^	0.27 ± 0.02 ^f^	28.7 ± 0.2 ^a^	T9B1	0.45 ± 0.01 ^b^	25 ± 1 ^b^	0.26 ± 0.01 ^j^	42.3 ± 0.1 ^c^
	T9L2	0.34 ± 0.01 ^b^	21 ± 1 ^g^	0.07 ± 0.01 ^c^	30.1 ± 0.1 ^a^	T9B2	0.44 ± 0.01 ^b^	30 ± 0 ^c^	0.02 ± 0.01 ^c^	33.0 ± 0.8 ^a^

Time: T0 (initial time), T1 (first month), T3 (third month), T6 (sixth month), T9 (ninth month). Wine: L—Loureiro monovarietal wine, B—Blend wine. Stage: 0—before transportation, 1—after transportation, 2—deoxygenation. Means within a column followed by the same letter are not significantly different according to Tukey’s test (*p* < 0.05). *n* = 3 experimental repetitions, 3 analytical repetitions.

**Table 2:** Kinetic model for dissolved oxygen, sulfur dioxide, and volatile acidity of the Loureiro monovarietal wine and Blend wine before and after transport.

Wine	Oxygen—Pseudo 2nd Order	k	r	*p*-Value <
Loureiro (L)	Stage 0	0.825 ± 0.036	0.983	0.0001
	Stage 1	0.372 ± 0.039	0.915	0.0001
	Stage 2	-	-	-
Blend (B)	Stage 0	0.115 ± 0.008	0.955	0.0001
	Stage 1	0.377 ± 0.014	0.988	0.0001
	Stage 2	-	-	-
	**Free sulfur dioxide—Pseudo 2nd Order**			
Loureiro (L)	Stage 0	0.00353 ± 0.00014 ^a^	0.987	0.0001
	Stage 1	0.00325 ± 0.00024 ^a^	0.955	0.0001
	Stage 2	0.00218 ± 0.00010	0.980	0.0001
Blend (B)	Stage 0	0.00081 ± 0.00011 ^a,b^	0.872	0.0001
	Stage 1	0.00118 ± 0.00024 ^a^	0.760	0.0001
	Stage 2	0.000510 ± 0.00012 ^b^	0.704	0.0005
	**Volatile Acidity—Pseudo Zero-Order**			
Loureiro (L)	Stage 0	0.0116 ± 0.0007 ^a^	0.969	0.0001
	Stage 1	0.0107 ± 0.0011 ^a^	0.911	0.0001
	Stage 2	0.00696 ± 0.00082	0.895	0.0001
Blend (B)	Stage 0	0.0157 ± 0.0010	0.967	0.0001
	Stage 1	0.0109 ± 0.0017 ^a^	0.834	0.0001
	Stage 2	0.0105 ± 0.0011 ^a^	0.913	0.0001

Stage 0—before transportation, Stage 1—after transportation, Stage 2—deoxygenation. Wine: L—Loureiro monovarietal wine, B—Blend wine. k—kinetic constant; r—correlation coefficient. For each wine, means within a column followed by the same letter are not significantly different *t*-Student test (*p* < 0.05).

**Table 3:** Color and chromatic characteristics of the Loureiro monovarietal wine and the Blend wine before and after transportation and deoxygenation over nine months (mean ± standard deviation).

		Color ^1^ (A420 nm)	L *	a *	b *	ΔE *		Color ^1^ (A420 nm)	L * (%)	a *	b *	ΔE *
	Loureiro monovarietal wine							Blend wine				
Initial time	T0L0	0.086 ± 0.002 ^a^	99.0 ± 0.0 ^a^	−2.09 ± 0.03 ^a^	6.57 ± 0.07 ^a^		T0B0	0.128 ± 0.001 ^a^	91.3 ± 0.0 ^a^	0.09 ± 0.01 ^a^	3.99 ± 0.03 ^a^	
	T0L1	0.085 ± 0.001 ^a^	97.9 ± 0.0 ^b^	−2.79 ± 0.00 ^a^	6.65 ± 0.00 ^a^	1.70 ± 0.11	T0B1	0.128 ± 0.010 ^a^	90.0 ± 0.2 ^b^	0.30 ± 0.12 ^b^	3.67 ± 0.02 ^a^	1.37 ± 0.02
	T0L2	0.080 ± 0.001 ^a^	97.2 ± 0.0 ^b^	−2.80 ± 0.01 ^a^	6.84 ± 0.04 ^a^	1.87 ± 0.04	T0B2	0.128 ± 0.001 ^a^	96.3 ± 0.0 ^c^	−2.28 ± 0.01 ^c^	4.74 ± 0.01 ^b^	1.86 ± 0.02
First month	T1L0	0.089 ± 0.004 ^a^	98.9 ± 0.0 ^c^	−1.81 ± 0.05 ^b^	7.17 ± 0.01 ^a^		T1B0	0.150 ± 0.004 ^a^	99.3 ± 0.1 ^d^	−1.58 ± 0.01 ^d^	4.09 ± 0.02 ^a^	
	T1L1	0.092 ± 0.002 ^a^	98.9 ± 0.0 ^c^	−2.04 ± 0.00 ^c^	7.26 ± 0.05 ^b^	1.98 ± 0.21	T1B1	0.142 ± 0.003 ^a^	99.6 ± 0.0 ^d^	−1.61 ± 0.03 ^d^	4.97 ± 0.02 ^b^	0.34 ± 0.06
	T1L2	0.085 ± 0.001 ^a^	98.9 ± 0.0 ^c^	−1.76 ± 0.04 ^b^	7.34 ± 0.20 ^b^	1.86 ± 0.01	T1B2	0.135 ± 0.001 ^a^	99.1 ± 0.3 ^d^	−1.59 ± 0.03 ^d^	4.30 ± 0.01 ^c^	0.45 ± 0.27
Third month	T3L0	0.126 ± 0.014 ^b^	97.9 ± 0.1 ^b^	−1.94 ± 0.03 ^c^	7.68 ± 0.16 ^b^		T3B0	0.156 ± 0.006 ^b^	98.6 ± 0.0 ^e^	−1.33 ± 0.02 ^e^	5.95 ± 0.01 ^d^	
	T3L1	0.129 ± 0.005 ^b^	98.5 ± 0.0 ^c^	−2.78 ± 0.02 ^a^	7.85 ± 0.06 ^b^	0.64 ± 0.04	T3B1	0.149 ± 0.004 ^a^	98.3 ± 0.0 ^e^	−1.64 ± 0.07 ^d^	4.28 ± 0.00 ^c^	0.72 ± 0.10
	T3L2	0.085 ± 0.001 ^a^	97.9 ± 0.0 ^b^	−1.97 ± 0.03 ^c^	7.19 ± 0.01 ^a^	0.28 ± 0.00	T3B2	0.145 ± 0.001 ^a^	98.4 ± 0.2 ^e^	−1.59 ± 0.01 ^d^	4.73 ± 0.00 ^b^	0.66 ± 0.00
Sixth month	T6L0	0.133 ± 0.006 ^b^	98.7 ± 0.0 ^c^	−1.94 ± 0.03 ^c^	7.92 ± 0.05 ^b^		T6B0	0.162 ± 0.003 ^c^	98.2 ± 0.0 ^e^	−1.57 ± 0.03 ^d^	6.74 ± 0.01 ^e^	
	T6L1	0.189 ± 0.009 ^c^	98.8 ± 0.0 ^c^	−2.78 ± 0.02 ^a^	8.86 ± 0.02 ^c^	1.84 ± 0.07	T6B1	0.158 ± 0.011 ^b^	97.5 ± 0.0 ^f^	−1.18 ± 0.01 ^f^	7.35 ± 0.07 ^f^	1.72 ± 0.11
	T6L2	0.110 ± 0.004 ^d^	98.6 ± 0.1 ^c^	−1.97 ± 0.03 ^c^	7.15 ± 0.03 ^a^	0.09 ± 0.00	T6B2	0.155 ± 0.002 ^b^	89.3 ± 0.0 ^b^	−1.13 ± 0.00 ^f^	6.27 ± 0.05 ^e^	8.98 ± 0.01
Ninth month	T9L0	0.166 ± 0.006 ^e^	98.9 ± 0.1 ^c^	−1.95 ± 0.02 ^c^	8.81 ± 0.01 ^c^		T9B0	0.167 ± 0.002 ^c^	97.6 ± 0.0 ^f^	−1.14 ± 0.00 ^f^	7.23 ± 0.02 ^f^	
	T9L1	0.193 ± 0.003 ^c^	99.0 ± 0.0 ^a^	−3.07 ± 0.04 ^d^	8.89 ± 0.02 ^c^	1.19 ± 0.09	T9B1	0.180 ± 0.003 ^d^	97.4 ± 0.0 ^f^	−1.17 ± 0.00 ^f^	9.40 ± 0.24 ^g^	0.70 ± 0.03
	T9L2	0.157 ± 0.001 ^e^	98.9 ± 0.0 ^c^	−2.07 ± 0.05 ^c^	7.16 ± 0.03 ^a^	0.60 ± 0.28	T9B2	0.156 ± 0.005 ^b^	89.2 ± 0.0 ^b^	−1.52 ± 0.07 ^d^	6.55 ± 0.04 ^e^	8.38 ± 0.02

Time: T0 (initial time), T1 (first month), T3 (third month), T6 (sixth month), T9 (ninth month). Wine: L—Loureiro monovarietal wine, B—Blend wine. Stage: 0—before transportation, 1—after transportation, 2—deoxygenation. L *—lightness, a *—redness, b *—yellowness, ΔE *—color difference. The values corresponding to ΔE * were obtained, taking as reference the wine before transport. ^1^—absorbance units. Means within a column followed by the same letter are not significantly different according to Tukey’s test (*p* < 0.05). *n* = 3 experimental repetitions, 3 analytical repetitions.

**Table 4:** Kinetic model for b * (yellowness) of the Loureiro monovarietal wine and Blend wine before and after transport.

Wine	Pseudo Zero-Order	k	r	*p*-Value <
Loureiro (L)	Stage 0	0.220 ± 0.020 ^a^	0.968	0.0001
	Stage 1	0.253 ± 0.030 ^a^	0.949	0.0001
	Stage 2	0.0122 ± 0.0278	0.247	0.689
Blend (B)	Stage 0	0.409 ± 0.045 ^a^	0.956	0.0001
	Stage 1	0.616 ± 0.068	0.955	0.0001
	Stage 2	0.256 ± 0.058 ^a^	0.931	0.0217

Stage 0—before transportation, Stage 1—after transportation, Stage 2—deoxygenation. Wine: L—Loureiro monovarietal wine, B—Blend wine. k—kinetic constant; r—correlation coefficient. For each wine, means within a column followed by the same letter are not significantly different *t*-Student test (*p* < 0.05).

**Table 5:** Phenolic compounds of the Loureiro monovarietal wine and the Blend wine before and after transportation and deoxygenation over nine months (mean ± standard deviation).

		Total Phenols (mg/L Gallic Acid)	Non-Flavonoid Phenols (mg/L Gallic Acid)	Flavonoid Phenols (mg/L Gallic Acid)	Gelatin Index (%)	Total Tannins (g/L)		Total Phenols (mg/L Gallic Acid)	Non-Flavonoid Phenols (mg/L Gallic Acid)	Flavonoid Phenols (mg/L Gallic Acid)	Gelatin Index (%)	Total Tannins (g/L)
	Loureiro Monovarietal Wine						Blend Wine					
Initial Time	T0L0	399 ± 1 ^a^	148 ± 1 ^a^	251 ± 1 ^a^	9 ± 0 ^a^	0.2 ± 0.0 ^a^	T0B0	558 ± 2 ^a^	226 ± 5 ^a^	332 ± 3 ^a^	3 ± 0 ^a^	0.4 ± 0.0 ^a^
	T0L1	397 ± 4 ^a^	153 ± 2 ^a^	244 ± 5 ^a^	9 ± 1 ^a^	0.2 ± 0.0 ^a^	T0B1	580 ± 6 ^a^	227 ± 5 ^a^	343 ± 7 ^a^	3 ± 0 ^a^	0.4 ± 0.0 ^a^
	T0L2	390 ± 1 ^b^	151 ± 1 ^a^	239 ± 2 ^b^	8 ± 1 ^b^	0.2 ± 0.0 ^a^	T0B2	513 ± 6 ^b^	228 ± 3 ^a^	285 ± 6 ^c^	3 ± 0 ^a^	0.4 ± 0.0 ^a^
First Month	T1L0	377 ± 4 ^b^	147 ± 1 ^a^	230 ± 3 ^b^	7 ± 2 ^c^	0.2 ± 0.0 ^a^	T1B0	502 ± 1 ^c^	181 ± 1 ^b^	321 ± 1 ^a^	1 ± 0 ^c^	0.4 ± 0.0 ^a^
	T1L1	370 ± 4 ^b^	145 ± 2 ^a^	225 ± 3 ^b^	8 ± 1 ^b^	0.2 ± 0.0 ^a^	T1B1	518 ± 4 ^b^	168 ± 2 ^b^	350 ± 4 ^b^	1 ± 0 ^c^	0.4 ± 0.0 ^a^
	T1L2	381 ± 4 ^a^	150 ± 3 ^a^	231 ± 4 ^b^	7 ± 0 ^d^	0.2 ± 0.0 ^a^	T1B2	451 ± 1 ^d^	175 ± 2 ^b^	276 ± 2 ^c^	2 ± 0 ^b^	0.4 ± 0.0 ^a^
Third month	T3L0	364 ± 4 ^c^	144 ± 1 ^a^	220 ± 4 ^c^	9 ± 2 ^a^	0.2 ± 0.0 ^a^	T3B0	508 ± 1 ^c^	177 ± 4 ^b^	331 ± 4 ^a^	1 ± 0 ^c^	0.4 ± 0.0 ^a^
	T3L1	367 ± 4 ^c^	141 ± 3 ^a^	226 ± 5 ^c^	8 ± 0 ^b^	0.2 ± 0.0 ^a^	T3B1	509 ± 6 ^c^	200 ± 4 ^c^	309 ± 6 ^a^	1 ± 0 ^c^	0.4 ± 0.0 ^a^
	T3L2	352 ± 2 ^c^	143 ± 1 ^a^	209 ± 3 ^c^	8 ± 0 ^b^	0.2 ± 0.0 ^a^	T3B2	491 ± 5 ^e^	217 ± 5 ^d^	274 ± 7 ^c^	1 ± 0 ^c^	0.4 ± 0.0 ^a^
Sixth month	T6L0	355 ± 2 ^c^	134 ± 5 ^b^	221 ± 4 ^c^	7 ± 0 ^d^	0.2 ± 0.0 ^a^	T6B0	487 ± 2 ^e^	162 ± 1 ^e^	325 ± 5 ^a^	1 ± 0 ^c^	0.4 ± 0.0 ^a^
	T6L1	360 ± 1 ^c^	137 ± 1 ^b^	223 ± 8 ^c^	8 ± 0 ^b^	0.2 ± 0.0 ^a^	T6B1	474 ± 4 ^e^	198 ± 5 ^c^	276 ± 1 ^c^	1 ± 0 ^c^	0.4 ± 0.0 ^a^
	T6L2	352 ± 5 ^c^	129 ± 4 ^b^	223 ± 8 ^c^	6 ± 1 ^d^	0.2 ± 0.0 ^a^	T6B2	479 ± 2 ^e^	205 ± 1 ^c^	274 ± 3 ^c^	1 ± 0 ^c^	0.4 ± 0.0 ^a^
Ninth month	T9L0	321 ± 1 ^d^	132 ± 4 ^b^	189 ± 2 ^d^	6 ± 1 ^d^	0.2 ± 0.0 ^a^	T9B0	441 ± 3 ^d^	149 ± 3 ^e^	292 ± 8 ^c^	1 ± 0 ^c^	0.4 ± 0.0 ^a^
	T9L1	359 ± 1 ^c^	132 ± 2 ^b^	227 ± 9 ^c^	7 ± 0 ^d^	0.2 ± 0.0 ^a^	T9B1	385 ± 6 ^f^	126 ± 1 ^f^	259 ± 8 ^d^	1 ± 0 ^c^	0.4 ± 0.0 ^a^
	T9L2	341 ± 4 ^c^	127 ± 5 ^b^	214 ± 5 ^c^	6 ± 0 ^d^	0.2 ± 0.0 ^a^	T9B2	445 ± 5 ^d^	186 ± 2 ^b^	259 ± 3 ^d^	1 ± 0 ^c^	0.4 ± 0.0 ^a^

Time: T0 (initial time), T1 (first month), T3 (third month), T6 (sixth month), T9 (ninth month). Wine: L—Loureiro monovarietal wine, B—Blend wine. Stage: 0—before transportation, 1—after transportation, 2—deoxygenation. Means within a column followed by the same letter are not significantly different according to Tukey’s test (*p* < 0.05). n = 3 experimental repetitions, 3 analytical repetitions.

**Table 6:** Kinetic model for total phenols of the Loureiro monovarietal and Blend wine before and after transport.

Wine	Pseudo Zero-Order	k	r	*p* <
Loureiro (L)	Stage 0	7.61 ± 0.57 ^a^	0.953	0.0001
	Stage 1	6.52 ± 1.14 ^a^	0.957	0.0001
	Stage 2	5.15 ± 1.39 ^a^	0.906	0.0342
Blend (B)	Stage 0	3.79 ± 0.39	0.917	0.0001
	Stage 1	6.56 ± 0.71 ^a^	0.909	0.0001
	Stage 2	6.38 ± 0.86 ^a^	0.977	0.0001

Stage 0—before transportation, Stage 1—after transportation, Stage 2—deoxygenation. Wine: L—Loureiro monovarietal wine, B—Blend wine; k—kinetic constant; r—correlation coefficient. For each wine, means within a column followed by the same letter are not significantly different *t*-Student test (*p* < 0.05).

## Data Availability

Not applicable.
